# Eloquent Extract

**Published:** 1853-10

**Authors:** 


*Eloquent Extract.—(Drake.)—(Scene—Fort Holmes.)*

When the observer directs his eye upon the water more than the land, and the day is fair, with moderate wind, he finds the surface as variable in its tints as if clothed in a robe of changeable silk. Green and blue are the governing hues, but they flow into each other with such facility and frequency, that while still contemplating a particular spot, it seems, as if by magic, transformed into another; but these mid-day beauties vanish before those of the setting sun, when the boundless horizon of lake and land seems girt round with a fiery zone of clouds, and the brilliant drapery of the skies paints itself upon the surface of the waters. Brief as they are beautiful, these evening glories, like spirits of the air, quickly pass away; and the gray mantle of night warns the beholder to depart for the village, while he may yet make his way along a narrow and rocky path, beset with tufts of prickly juniper. Having refreshed himself for an hour, he may stroll out upon the beach to listen to the serenade of the waters. Wave after wave will break at his feet, over the white pebbles, and return as limpid as it came. Up the straits he will see the evening star dancing on the ruffled surface, and the loose sails of the lagging schooner flapping in the fitful land breeze; while the milky way, Death's Path of the red man, will dimly appear on the waters before him. Behind, in the street, a lively group of Canadian French, of every shade of color between white and red, will gossip and shrug their shoulders; on one side, should the Indians, who still inhabit the shores of Lake Michigan, be on a visit to the island, he will hear the uproar of a lodge of drunken Chippeways, with the screams of women and children, and the cackling of frightened hens; on the other, will see the sober and listless Ottawa, sitting in silent vacancy of thought, on his upturned birch canoe; his wife within the tent, spreading cypress bark and flag mats upon the gravel, as lodgings for the night; while half a dozen children loll or play about the door, and as many half-starved dogs curl up among them. Surrounded by such scenes, the traveller begins to realize that he is a stranger; when suddenly, a new phenomenon appears and fixes the conviction. Every object becomes more visible; and, raising his eyes, he beholds the heavens illuminated with an aurora borealis, where he reads, in fantastic characters of strange and eccentric light, that he is, indeed, a sojourner in a strange land, and has wandered far from home and his friends in the sunny regions of the South.—*Dr. Meigs' Notice of Dr. Drake. p.* 28.

